# Thin flap sulcus‐deepening trochleoplasty in patellar instability yields good functional outcomes without progressive cartilage deterioration in the short‐term follow‐up—A retrospective single‐surgeon cohort study

**DOI:** 10.1002/ksa.12566

**Published:** 2024-12-25

**Authors:** Jannik Frings, Eva Janssen, Matthias Krause, Karl‐Heinz Frosch, Eik Vettorazzi, Andreas Weiler, Arno Schmeling

**Affiliations:** ^1^ Department of Trauma and Orthopaedic Surgery University Medical Center Hamburg‐Eppendorf Hamburg Germany; ^2^ Department of Trauma Surgery, Orthopedics and Sports Traumatology BG Hospital Hamburg Hamburg Germany; ^3^ Institute of Medical Biometry and Epidemiology University Medical Center Hamburg‐Eppendorf Hamburg Germany; ^4^ Sporthopaedicum Berlin Germany

**Keywords:** cartilage deterioration, maltracking, patellofemoral instability, trochlear dysplasia, trochleoplasty

## Abstract

**Purpose:**

Sulcus‐deepening trochleoplasty (TP) effectively treats patellofemoral (PF) instability (PFI) caused by high‐grade trochlear dysplasia (TD), but current evidence is based on small case series. We hypothesised, that TP would result in significant functional improvements and a low re‐dislocation rate but would not accelerate the progression of PF cartilage deterioration.

**Methods:**

We retrospectively reviewed all TP cases performed by a single surgeon between 2015 and 2021. Inclusion criteria were postoperative Magnetic resonance imaging (MRI) >6 and >12 months and a clinical follow‐up >12 months. Patients with simultaneous cartilage repair, open physes or incomplete records were excluded. Clinical outcomes were assessed using pre‐ and postoperative scores, postoperative Banff Patellofemoral Instability Instrument (BPII) 2.0 and Knee Injury and Osteoarthritis Outcome Score (KOOS), re‐dislocation rate and patient satisfaction. PF cartilage was evaluated via Area Measurement and Depth & Underlying Structures (AMADEUS) scores preoperatively, at 6 months and at the final follow‐up.

**Results:**

We included 113 patients (25.8 ± 8.3 years) with high‐grade TD (Dejour B–D; mean lateral inclination angle: −2.9 ± 9.1°), 85% of whom had advanced cartilage lesions. All underwent TP, lateral retinacular lengthening (LRL) and medial patellofemoral ligament reconstruction (MPFL‐R). After 34.8 ± 20.9 months, function, pain levels and Tegner scores improved significantly (*p* < 0.001). KOOS dimensions were: symptoms 79.9 ± 13.5, pain 86.4 ± 12.1, activity 91.9 ± 8.3, sports 71.7 ± 22.2 and quality‐of‐life 58.1 ± 23.8. BPII 2.0 was 64.3 ± 31.4. Preoperative AMADEUS scores (55.2 ± 17.4) remained stable at 6 months (*p* = 0.343) but improved to 58.4 ± 16.0 at 28.6 (12–89) months (*p* = 0.004). Complication and re‐dislocation rates were 5.3% and 1.8%, with 93% patient satisfaction.

**Conclusion:**

Sulcus‐deepening TP with MPFL‐R and LRL yields good to excellent short‐term results without progressive chondral deterioration, enabling patients to return to their prior or higher activity levels despite advanced preoperative chondral lesions. TP can be considered a safe, joint‐preserving technique for PF stabilisation.

**Level of Evidence:**

Level III, retrospective cohort study.

## Graphical abstract

Thin‐flap sulcus‐deepening trochleoplasty with medial patellofemoral ligament reconstruction is a safe and effective procedure in patellar instability and high‐grade trochlear dysplasia, with no cartilage deterioration after 34.8 ± 20.9 months.

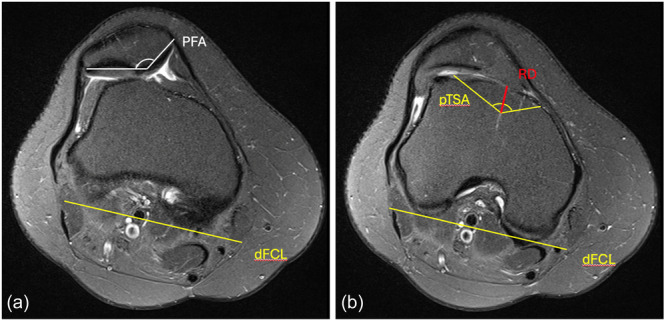

**Abbreviations**
AMADEUS: Area Measurement and Depth & Underlying Structures
BMI: body mass index
BPII: Banff Patellofemoral Instability Instrument
CDI: Caton–Deschamps index
CI: confidence interval
dFCL: dorsal femoral condylar line
ICC: intraclass correlation coefficient
ICRS: International Cartilage Repair Society
ISR: Insall–Salvati ratio
KOOS: Knee Injury and Osteoarthritis Outcome Score
LRL: lateral retinacular lengthening
LTI: lateral trochlear inclination angle
MIC: minimal clinically important change
MPFL: medial patellofemoral ligament
MPFL‐R: medial patellofemoral ligament reconstruction
MR: magnetic resonance
MRI: magnetic resonance imaging
PD FSE: proton density fast spin echo
PF: patellofemoral
PFA: patellar facet angle
PFI: patellofemoral instability
PFJ: patellofemoral joint
PF‐OA: patellofemoral osteoarthritis
PM: patellar maltracking
pTSA: planned trochlear sulcus angle
QoL: quality of life
RD: resection depth
RF: risk factor
TD: trochlear dysplasia
TP: trochleoplasty
TT‐PCL: tibial tubercle to posterior cruciate ligament
VAS: visual analogue scale

## INTRODUCTION

Trochlear dysplasia (TD) is one of the most influential risk factors (RF) for patellofemoral (PF) instability [22, 45]. Especially in severe cases, such as Dejour types B–D, patellar tracking is misguided by a convex sulcus configuration, causing relevant lateralisation or the total loss of stabile PF articulation (patellofemoral maltracking; PM) [23, 25]. Several technical variations of trochleoplasty (TP) have been described, aiming to restore a physiological shape of the trochlear groove [8, 18, 24]. Among them, Bereiter's thin‐flap, sulcus‐deepening technique is one of the most common and frequently used techniques [8, 35]. Recent literature mostly reports good to very good short‐term clinical results, considering significant improvements in Kujala, Tegner and International Knee Documentation Committee (IKDC) scores, as well as low recurrence rates at 2 years [6, 7, 15, 27, 42]. However, most studies report on comparably small case series or include short follow‐up periods [7, 34, 42, 47]. Other studies with larger case series have reported limited functional improvements, revision rates of 14%–19.6%, and persistent instability in 8.3%–9.3% at 6 and 11 years, respectively, especially when TP was not combined with medial patellofemoral ligament reconstruction (MPFL‐R) [37, 40]. Besides these clinical considerations, concerns have been raised, that TP may lead to the progression of cartilage deterioration or even cause patellofemoral osteoarthritis (PF‐OA) in the mid‐ to long‐term follow‐up, with reported incidences of PF‐OA as high as 27% following TP [35, 36, 48]. At the same time, there is a high variability in reported functional outcomes and complication rates in the literature depending on the different techniques for TP, which were often combined with additional bony realignment procedures [36, 55].

The aim of this study was, to evaluate the clinical outcome after sulcus‐deepening TP combined with MPFL‐R. We hypothesised that TP with MPFL‐R would result in significant functional improvements and a low re‐dislocation rate. It was also hypothesised that TP with MPFL‐R would not be associated with patellofemoral cartilage deterioration.

## MATERIALS AND METHODS

### Patient selection

Patients with PFI and PM due to high‐grade TD who underwent TP by the senior author between 2015 and 2021 were identified and their medical records were retrospectively reviewed. Patients from this period were included if they were treated with TP, if pre‐ and postoperative magnetic resonance (MR) images with a minimum follow‐up of 6 (first radiologic end point) and 12 months (second radiologic end point) were present, and if written informed consent was given. A minimum follow‐up of 12 months was required for clinical evaluation. Exclusion criteria were incomplete medical records, open physes, concomitant cartilage repair, or relevant extra‐articular RFs, such as torsional or valgus leg axis deformities, patella alta (Caton–Deschamps index [CDI] > 1.2) or a tibial tuberosity to posterior cruciate ligament (TT‐PCL ≥ 25 mm). Additional cartilage repair was only performed in an acute setting and focal defect configuration, but not in the patient collective of this study. Patients were also excluded if no follow‐up MR images were available or if the minimum time for the clinical or radiologic follow‐up was not reached. The study design was approved by the local ethics committee (ID PV5191) and was conducted in accordance with the Good Clinical Practice Guidelines and the tenets of the Declaration of Helsinki.

### Functional evaluation

Functional outcome parameters were prospectively collected. Eligible patients were asked to complete an outcome questionnaire with standardised functional outcome scores, pre‐ and postoperatively. Pain was assessed using a visual analogue scale (VAS) and Kujala's anterior knee pain score. Tegner activity index was used to assess patients' activity levels, and the Lysholm score, Knee Injury and Osteoarthritis Outcome Score (KOOS) and the Banff Patellofemoral Instability Instrument (BPII) 2.0 were used to assess knee function and quality of life. Re‐dislocation and complication rates were assessed at the final follow‐up. General patient satisfaction was assessed by asking patients if they would choose the procedure again (yes or no).

### Radiological evaluation

Common RF for patellofemoral instability (PFI), such as TT‐PCL distance [53], patellar tilt, CDI and Insall–Salvati ratio (ISR) [11, 30], were measured on preoperative MR images. TD was characterised on transverse MR images, using the Dejour classification [16]. In addition, the lateral trochlear inclination angle (LTI) was measured on transverse slices at the level of the most proximal extent of the trochlear cartilaginous surface [10]. The quantitative and qualitative appearance of patellofemoral cartilage was analysed on transverse proton density fast spin echo (PD FSE) MRI sequences acquired preoperatively, 6 months and at least 12 months post‐operatively using the Area Measurement and Depth & Underlying Structures (AMADEUS) score and classification [32]. The radiologic evaluation was performed by a fellowship‐trained sports trauma specialist and a sports trauma resident. Intraclass correlation coefficient (ICC) and corresponding 95% confidence interval (CI) were calculated to test intra‐ and interrater agreement.

### Surgical indication and technique

**Figure 1:**
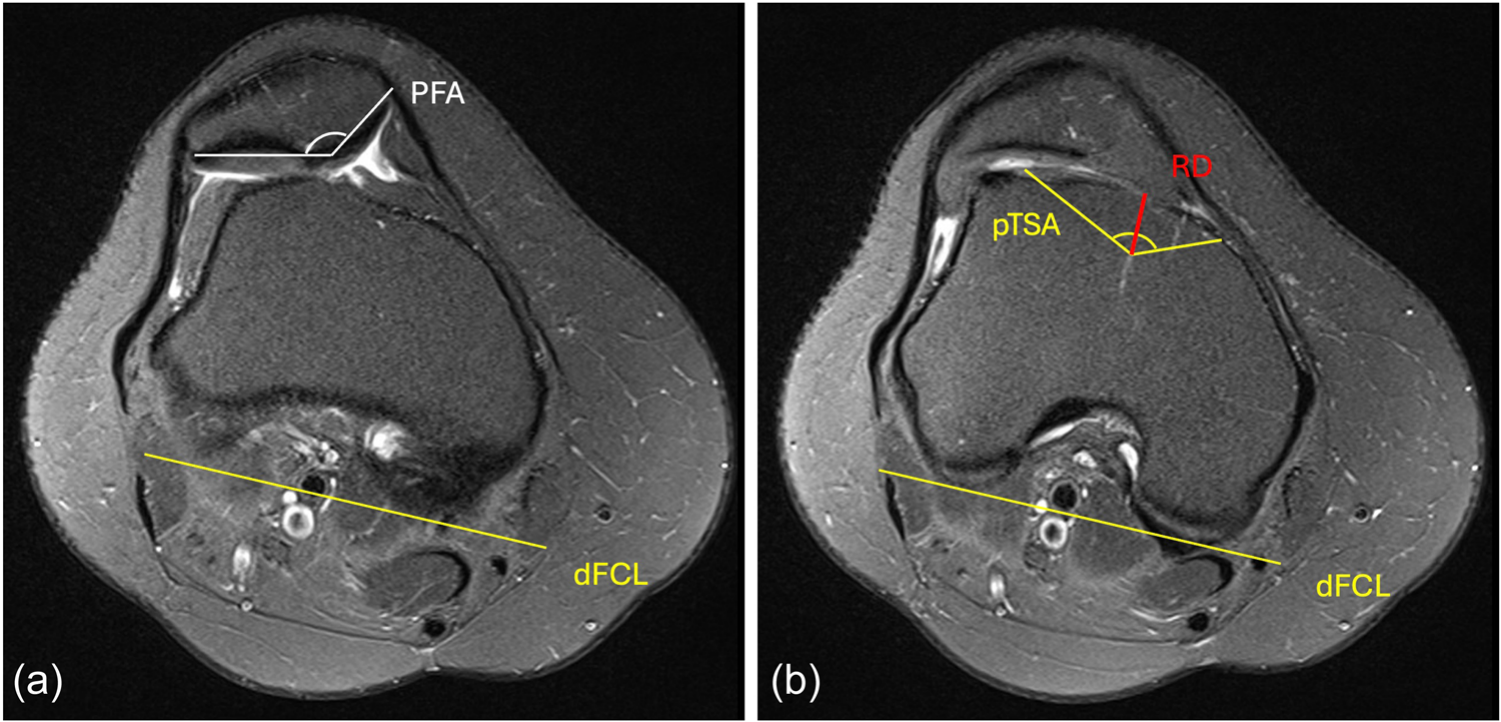
Preoperative planning of trochleoplasty. The planned trochlear sulcus angle (pTSA) was designed according to the patellar facet angle (PFA) so that the pTSA corresponded to the PFA and was related to the dorsal femoral condylar line (dFCL) (a). The resection depth (RD) was determined according to the maximum femoral offset. In this case, a pTSA of 145° was targeted, resulting in a planned resection depth of 8 mm, measured from the most anterior part of the femoral offset (proximal to this slice) (b).

TP was indicated in patients who presented with recurring patellar dislocations, in combination with PM, caused by high‐grade TD (Dejour types B–D) [43]. Under these conditions, an LTI of <11°, a supratrochlear bump and an increased femoral offset were considered for the indication [10]. All TP were performed by the senior author, using Bereiter's thin‐flap, sulcus‐deepening technique [8]. The procedures were preoperatively planned, on PD FSE MR images in transverse and sagittal orientation (Figure 1a). In this regard, the aspired sulcus angle and resection depth were determined in relation to the individual shape of the patella (Figure 1b). PF tracking and articular cartilage were arthroscopically evaluated, according to the International Cartilage Regeneration & Joint Preservation Society (ICRS).

Management of chondral lesions followed the concept of ‘unloading’ the affected areas by performing trochleoplasty. There were no additional cartilage repair procedures in this study.

**Figure 2:**
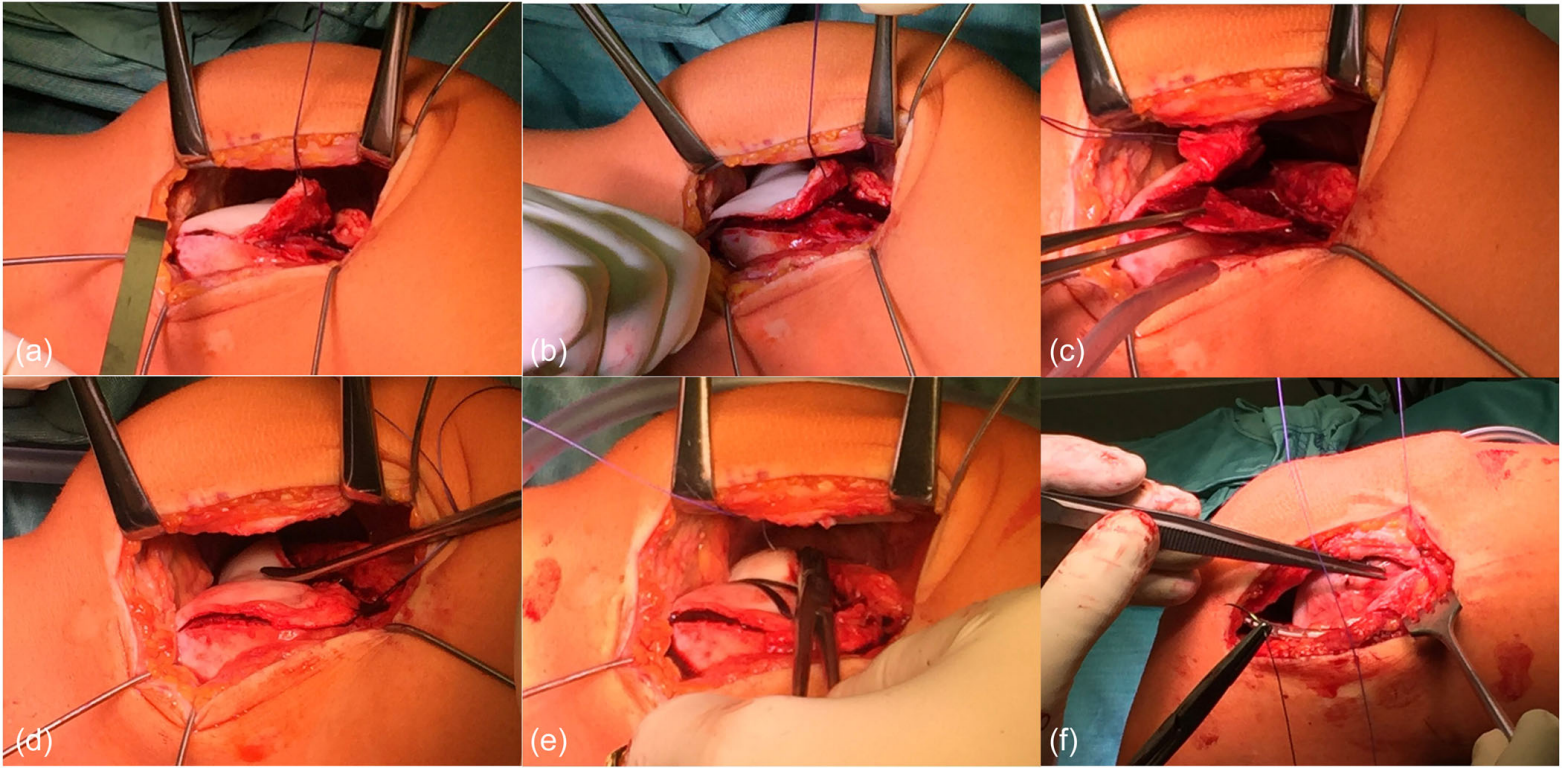
Thin‐flap sulcus‐deepening trochleoplasty. A periostal flap was developed (a), followed by a subchondral osteotomy (b). Dysplastic subchondral bone was removed according to the preoperative plan (c) to create a new groove (d). The reduced chondral flap was refixed with 3 mm Vicryl tape (e), followed by lateral retinacular lengthening.

Patients were placed in supine position. A lateral parapatellar approach was developed and the lateral retinaculum was incised in a Z‐shaped fashion, allowing for subsequent lateral retinaculum lengthening (LRL). After exposure to the dysplastic trochlea, a thin osteochondral flap was created with osteotomes (Figure 2a,b). The location of the new trochlear groove was determined in relation to the shape of the patella and according to physiological anatomy. The sulcus was formed by subsequent resection of excess bone (Figure 2c), confirmed by measurement of the resection depth. Finally, the osteochondral flap was reduced, moulded into the groove (Figure 2d), and tensioned with a 3 mm Vicryl tape, which was fixed in a V‐shaped fashion using three 3.5 × 19.5 mm^2^ PushLock® (Arthrex) anchors (Figure 2e). The lateral retinaculum was closed in 70° of knee flexion, allowing for a retinacular lengthening (Figure 2f).

All TP were complemented by MPFL‐R, using an ipsilateral gracilis autograft, which was fixed to the patella with two suture anchors (SutureTak® 3 mm, Arthrex or BioPlug® 3,5 mm, Karl Storz) and into the femoral attachment with a 6 mm interference screw, after fluoroscopic identification [51].

### Statistical analysis

Data collection and explorative analysis were performed using Excel, version 16.31 (Microsoft). Calculations were performed using SPSS Statistics, version 21.0 (IBM). Response data were presented as means and standard deviations. Differences in the preoperative and postoperative outcome data and AMADEUS score and classification were analysed using paired t‐tests, and pre‐post differences and CIs were reported. The significance level was set at *p* < 0.05. Spearman's correlation was calculated between the AMADEUS score, the body mass index (BMI), patient age at surgery and the postoperative functional outcome scores. Univariate analysis of covariance (ANCOVA) was used to examine the postoperative quality of life (QoL) subscale of the KOOS, including for previous surgery, patient age at the time of surgery, preoperative AMADEUS and Kujala scores as predictors.

## RESULTS

**Figure 3:**
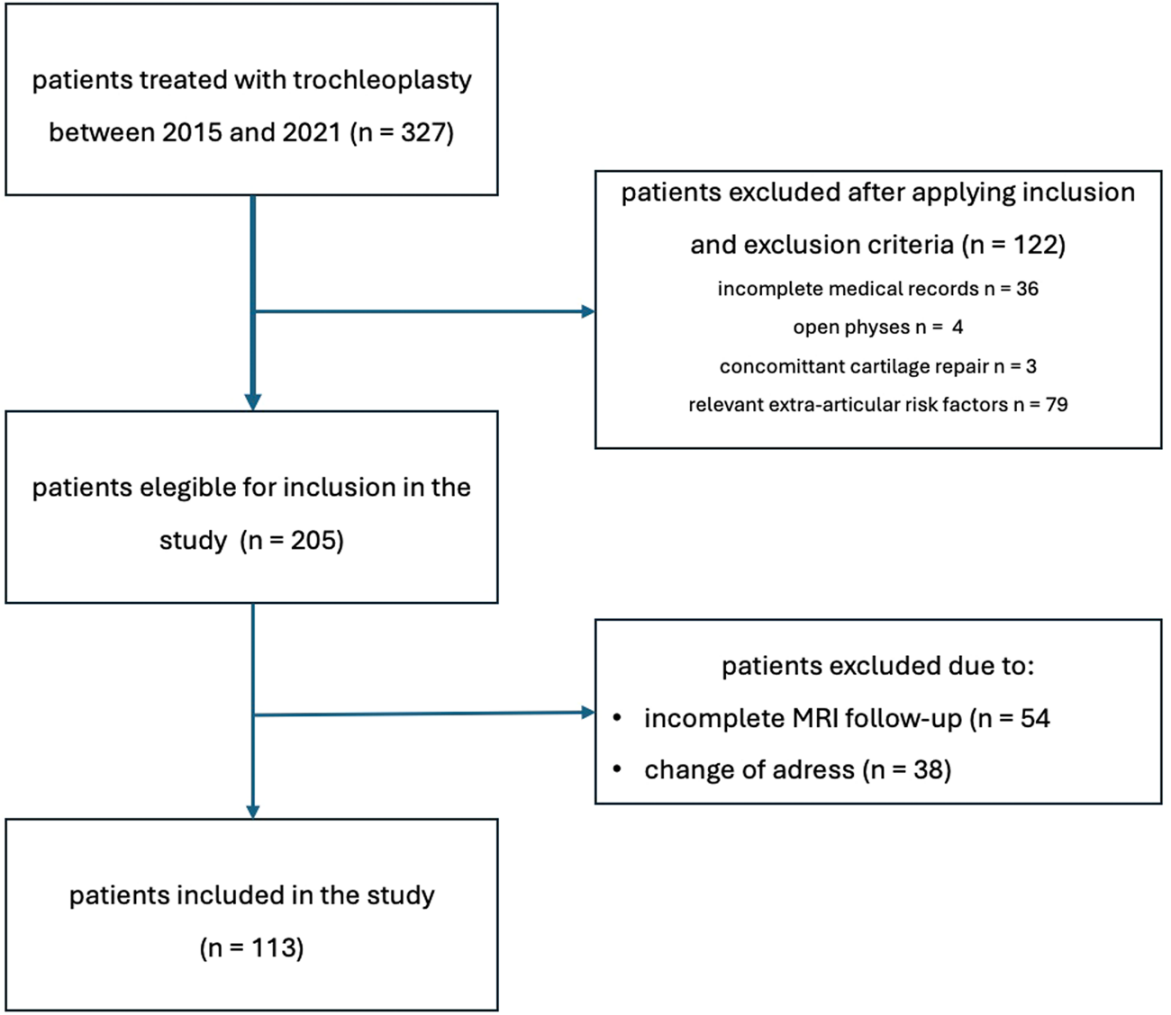
Flowchart on the composition of the patient collective.

Between 2015 and 2021, 327 patients with PFI and PM due to high‐grade TP were treated by the senior author. After applying inclusion and exclusion criteria, 122 patients were excluded due to incomplete medical records (*n* = 36), concomitant cartilage repair (n = 3), open physes (n = 4) or relevant extra‐articular RFs (*n* = 79) (Figure 3). A total of 205 patients were included, in the study. Ninety‐two patients (44.9%) were loss to follow‐up due to unknown change of address or were unable to provide a complete postoperative MRI follow‐up, leaving 113 patients (86 females, 27 males, average age 25.8 ± 8.3 [16–54] years) with a mean clinical follow‐up of 34.8 ± 20.9 (12–97) months and a mean radiologic follow‐up of 28.6 ± 18.9 (12‐89) months, including 6‐month MRI (Figure 3). Detailed patient characteristics are presented in Table 1.

**Table 1:** Patient characteristics and descriptive statistics of the included study collective, including anatomic risk factors.

Parameter	
Gender	Males = 27
	Females = 86
Age	25.8 ± 8.3 (16–54) years
BMI	24.6 ± 4.8
Follow‐up period	34.8 ± 20.9 (median 29) months
Previous surgeries	Yes = 75 (66.4%)
	No = 38 (33.6%)
	Number of previous surgeries = 1.21 (range 0–4)
	*Types of surgery*
	Medial reefing *n* = 32
	Lateral release *n* = 13
	MPFL‐R *n* = 10
	Goldthwait procedure *n* = 1
	Tibial tubercle osteotomy *n* = 6
	loose body removal *n* = 8
	varus producing osteotomy *n* = 4
	femoral de‐rotation osteotomy *n* = 1
Dejour types	*B* = 5 (4.4%)
	C = 57 (50.4%)
	*D* = 51 (45.1%)
Lateral trochlea inclination angle	−2.9 ± 9.1°
Patella tilt	25.3 ± 10.1°
TT‐PCL	20.4 ± 4.3 mm
Caton–Deschamps Index	1.1 ± 0.2
Insall–Salvati ratio	1.3 ± 0.2

*Note*: Data are presented as mean and standard deviations.

Abbreviations: BMI, body mass index; TT‐PCL, tibial tubercle to posterior cruciate ligament distance.

### Functional outcome

**Figure 4:**
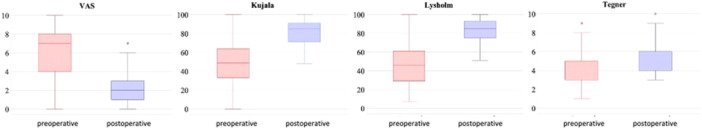
Preoperative versus postoperative functional outcome parameters, indicating significant improvements after the surgery.

**Figure 5:**
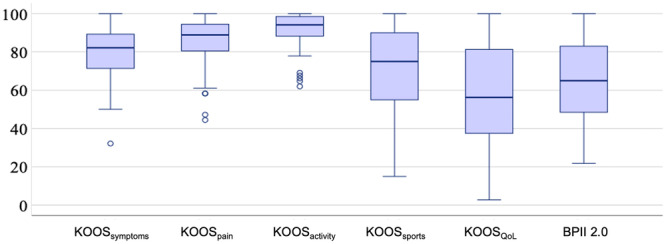
Postoperative KOOS and BPII 2.0 after 34.8 ± 21 months, showing good to excellent results.

At 34.8 ± 20.8 months, pain levels were significantly reduced and there was a significant increase in functional outcome parameters (Figure 4). There were good to excellent results in all KOOS dimensions except QoL and BPII 2.0 at the final follow‐up (Figure 5, Table 2). Overall patient satisfaction at final follow‐up was 93%.

**Table 2:** Presentation of PROMs and their changes from preoperative baseline to final follow‐up, along with postoperative outcomes for KOOS and BPII 2.0.

Functional outcome measures	Preoperative	Postoperative	Mean individual change (95% CI)	*p*
VAS pain	6.2 ± 2.8	1.9 ± 1.7	−4.1 (−4.6 to −3.6)	<0.001
Kujala anterior knee pain score	49.5 ± 21.8	80.6 ± 13.6	31.1 (26.7–35.6)	<0.001
Lysholm score	46.2 ± 20.6	83.0 ± 12.8	36.9 (32.9–40.8)	<0.001
Tegner activity index	3.8 ± 2.0	4.7 ± 1.6	0.9 (0.5–1.3)	<0.001
KOOS				
Symptoms	79.9 ± 13.5			
Pain	86.4 ± 12.1			
Activity	91.1 ± 8.3			
Sports/Rec	71.7 ± 22.2			
Quality of life	58.1 ± 23.8			
BPII 2.0	64.3 ± 21.4			

*Note*: Data are presented as mean and standard deviations.

Abbreviations: BPII 2.0, Banff Patella Instability Instrument 2.0; CI, confidence interval; KOOS, Knee Injury and Osteoarthritis Outcome Score; PROM, patient‐reported outcome measure; VAS, visual analogue scale.

With increasing age at the time of surgery, we observed an increase in PF cartilage lesions, both preoperatively (ICRS: *F* = 0.310, *p* = 0.001; AMADEUS preoperative: *F* = −0.235, *p* = 0.012) and postoperatively (AMADEUS at 6 months: *F* = −0.232, *p* = 0.019; AMADEUS final: *F* = −0.236, *p* = 0.039). Finally, a lower final AMADEUS score was correlated with a lower QoL dimension of the KOOS (*F* = 0.291, *p* = 0.010). All of the postoperative outcome parameters correlated with the postoperative QoL subscale of the KOOS, indicating a higher quality of life with improved knee function and decreased pain levels (*p* < 0.001). ANCOVA revealed a moderate effect of the preoperative Kujala score on the postoperative QoL subscale (*F* = 4.503; df = 1.108; *p* = 0.036), but no effect of all other tested variates

Within the study collective, a subgroup analysis was performed, including all patients with a minimum clinical follow‐up of 24 months (Table 3).

**Table 3:** Patient characteristics and descriptive statistics of a subgroup with a minimum follow‐up of 24 months, including anatomic risk factors.

Parameter	Patients included (*n* = 68)
Gender	Males = 12
	Females = 56
Age	25.1 ± 8.4 (14–54) years
BMI	23.8 ± 4.0
Follow‐up	46.5 ± 19.2 months
Previous surgeries	Yes = 45 (66.2%)
	No = 23 (33.8%)
	Number of previous surgeries = 1.35 (range 0–4)
	*Types of surgery*
	Medial reefing *n* = 20
	Lateral release *n* = 10
	MPFL‐R *n* = 6
	Goldthwait procedure *n* = 1
	Tibial tubercle osteotomy *n* = 3
	Loose body removal *n* = 4
	Varus‐producing osteotomy *n* = 1
Dejour types	*B* = 1 (1.5%)
	*C* = 39 (57.4%)
	*D* = 28 (41.2%)
Lateral trochlea inclination angle	−4.5 ± 9.5°
Patella tilt	27.4 ± 9.8°
TT‐PCL	21.9 ± 3.9 mm
Caton–Deschamps Index	1.1 ± 0.2
Insall–Salvati ratio	1.2 ± 0.2

*Note*: Data are presented as mean and standard deviations.

Abbreviations: BMI, body mass index; MPFL‐R, medial patellofemoral ligament reconstruction; TT‐PCL, tibial tubercle to posterior cruciate ligament distance.

At 46.5 ± 19.2 (25–97) months, 68 patients reported a significant reduction in pain and improvement in knee function as measured by Kujala, Lysholm and Tegner scores (*p* < 0.001). The functional outcome and quality of life measured by the KOOS dimensions and BPII were good to excellent (Table 4). The overall satisfaction rate was 91.2% and 61 patients would undergo the same procedure again.

**Table 4:** Presentation of PROMs and their changes from preoperative baseline to final follow‐up in patients with a minimum follow‐up of 24 months, along with postoperative outcomes for KOOS and BPII 2.0.

Functional outcome measures	Preoperative	Postoperative	Mean individual change (95% CI)	*p*
VAS pain	6.4 ± 2.6	1.9 ± 1.8	−4.4 (−5.0 to −3.8)	<0.001
Kujala's anterior knee pain score	48.3 ± 21.8	80.7 ± 14.8	32.4 (26.3–38.3)	<0.001
Lysholm score	46.1 ± 21.4	82.7 ± 13.8	36.3 (13.5–31.2)	<0.001
Tegner activity index	4.0 ± 2.0	4.9 ± 1.8	0.9 (0.5–1.4)	<0.001
KOOS				
Symptoms	79.7 ± 14.3			
Pain	86.9 ± 12.3			
Activity	92.5 ± 8.5			
Sports/Rec	72.8 ± 22.9			
Quality of life	58.2 ± 25.4			
BPII 2.0	64.4 ± 21.7			

*Note*: Data are presented as mean and standard deviations.

Abbreviations: BPII 2.0, Banff Patella Instability Instrument 2.0; CI, confidence interval; KOOS, Knee Injury and Osteoarthritis Outcome Score; PROM, patient‐reported outcome measure; VAS, visual analogue scale.

### Radiologic outcome

**Figure 6:**
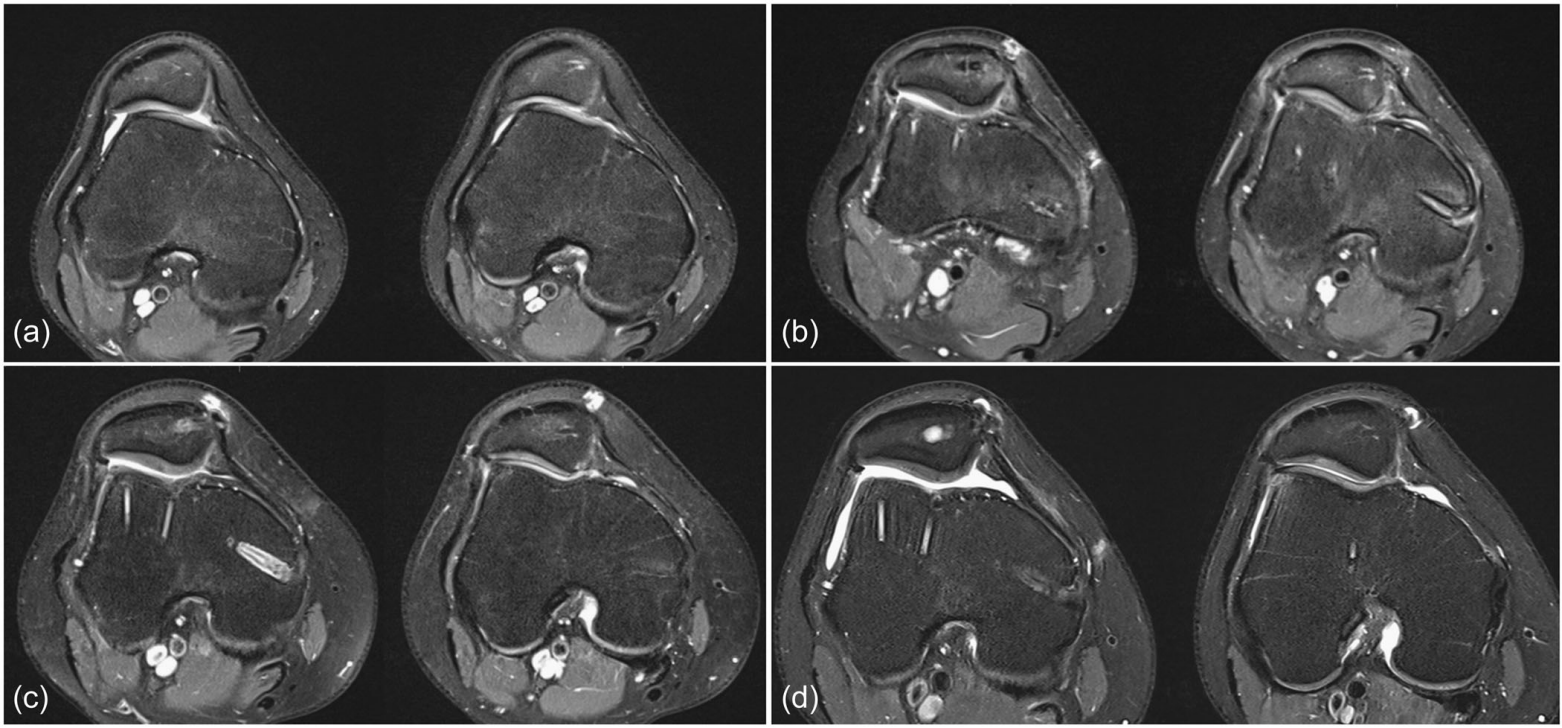
Patellofemoral cartilage evolution in a 14‐year‐old female patient. Initial magnetic resonance imaging (MRI) showed high‐grade dysplasia and signs of cartilage thinning at the lateral patellar facet (a). Eighteen months after trochleoplasty and MPFL‐R, patellofemoral congruence was improved (b). Follow‐up MRI at 35 months (c) and 112 months (d) showed an intact patellofemoral articular cartilage with no evidence of further degeneration. MPFL‐R, medial patellofemoral ligament reconstruction.

At the time of surgery, 95 knees (84.8%) presented ICRS Grades III and IV. patellofemoral cartilage lesions (77 [68.1%] Grade III, 18 [15.9%] Grade IV). The preoperative AMADEUS score was 55.2 ± 17.4. At 6 months post‐operatively, no significant changes in the AMADEUS score were observed (53.9 ± 16.7; difference 0.4, 95% CI = −0.5 to 1.3; *p* = 0.343). However, after 28.6 ± 18.9 (12–89) months (median 23 months), the AMADEUS score had significantly improved to 58.4 ± 16.0 (difference 3.2, 95% CI = 1.1–5.4; *p* = 0.004) (Figure 6).

**Figure 7:**
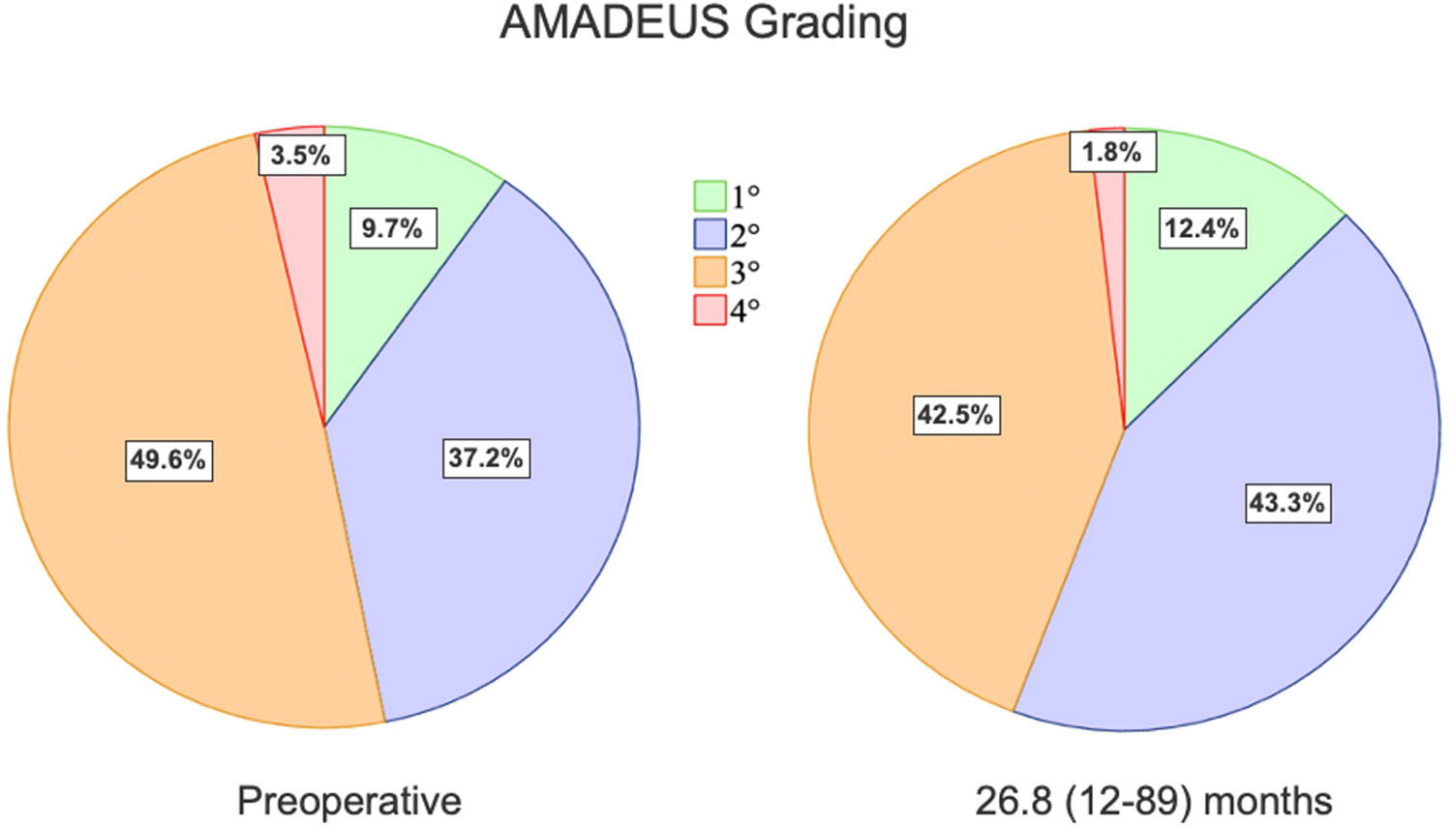
AMADEUS grading preoperatively and at final follow‐up. After 28.6 (12–89) months, no significant changes were observed. AMADEUS, Area Measurement and Depth & Underlying Structures.

Correspondingly, individual analyses using the AMADEUS grading showed no significant changes in 90 knees (79.6%), at final follow‐up. However, in 19 cases (16.8%), the AMADEUS grading had improved by an average of one degree, while in 4 cases (3.5%), the chondral lesion had worsened by one degree at the final radiologic follow‐up (Figure 7). Intra‐ and interrater agreement was very good (ICC = 0.94, 95% CI = 0.89–0.97 and ICC = 0.91, 95% CI = 0.83–0.95).

### Complications

Besides a re‐dislocation rate of 1.8% (*n* = 2), the surgery‐related complication rate at the final follow‐up was 5.3%, including one postoperative hemarthrosis (0.8%) and five cases with a restricted range of motion due to post‐operative stiffness (4.4%). All cases were successfully treated with minor arthroscopic revision.

## DISCUSSION

The most important finding of this study was that in the presence of high‐grade TD (Dejour B‐D), TP and MPFL‐R led to a significant reduction of pain and an improvement of functional outcome parameters, with a low re‐dislocation rate despite the high prevalence of pre‐existing cartilage lesions in this collective.

High‐grade TD is characterised by a reduced PF contact area and joint congruency, which compromises patellofemoral tracking, increases patellofemoral contact pressures, and predisposes the patellofemoral joint (PFJ) to instability and OA [12, 25, 28].

In a radiologic analysis, Balcarek et al. found that PFJ congruency was significantly improved by TP, compared to preoperative measurements [3]. The same group reported on functional outcomes of a series of 112 TPs with additional realignment procedures. They found that after 39.2 ± 9.9 months almost two‐thirds of patients had returned to their previous levels of activity [39]. Ng et al. investigated a case series of 58 combined TPs, which were combined with MPFL‐R in 93% and additional tibial tubercle transfer in 47% of all cases [44]. At a median follow‐up of 37 months, they observed a re‐dislocation rate of 5% [44]. This was in line with the findings of our study, which confirmed significant improvements in all functional outcome parameters, following TP. The comparatively lower re‐dislocation rate in our study is most likely due to the fact that additional MPFL‐R and LRL were performed in all cases. This also corresponds to the current evidence, according to which the combination of MPFL‐R leads to significantly better results in this context [46].

Although all functional parameters showed good to excellent results, the postoperative BPII 2.0. averaged 64/100 points, indicating a moderate functional outcome. In a comparable study, Ng et al. reported a mean BPII 2.0 score of 58.4/100 points, while the mean VAS score was only 30/100, respectively [44]. Balcarek et al. observed a BPII 2.0 score of 80.4 ± 16.9/100 points, but a high return‐to‐activity rate after 39 months [39]. In a case series of 16 arthroscopic TPs, Blond and Barfod found a BPII 2.0 score of 74.3/100 points, but excellent outcomes for the KOOS and Kujala scores [9]. Overall, the literature shows a certain incoherence of the BPII 2.0 with regard to the correlation with alternative outcome parameters. This could be explained by differences among the patient populations, surgical techniques, degree and amount of cartilage lesions or even rehabilitation protocols. However, there is also reasonable doubt as to whether a score of 100 is a realistically achievable benchmark so, consequently, the physiological reference value may be well below 100 points. In addition, patient age and preoperative score values are known to influence the likelihood of achieving the minimal clinically important change (MIC) of this score [14]. It has also been shown that the subjective disease‐specific quality of life is negatively influenced by a high grade of PF maltracking, as such is caused by TD [41]. In this study, we also found a moderate QoL score, suggesting a low QoL due to limited activity levels and persistent pain. At the same time, the remaining functional outcome parameters showed good to excellent results. According to a meta‐analysis, the MIC and relevance of the KOOS dimensions may vary depending on the investigated study collective. In this regard, Sport/Rec was considered the most important dimension in young patients with knee injuries, while Pain was the most relevant in painful knee conditions [13]. Ingelsrud et al. found that Sport/Rec and QoL were the most important subscales following anterior cruciate ligament reconstruction [29]. No such recommendation exists for the evaluation of PFI or TP, respectively. It is certainly possible that the moderate QoL reflects an insufficient patient‐perceived improvement in terms of limited activity level and persistent anterior knee pain. On the other hand, both aspects were significantly improved at the final follow‐up and we observed a high subjective patient satisfaction rate. Furthermore, a recent study found that adolescents with a history of patellar dislocation generally presented with reduced quality of life and function (BPII 2.0 and Kujala) but high activity levels compared to the general population [21]. In general, current evidence suggests good to excellent functional outcomes after TP at both short‐ and long‐term follow‐ups, almost regardless of the surgical technique used [17, 19, 20, 54]. At the same time, high incidences of PF‐OA (26%–30%) have been reported, 4–8 years after TP [34, 35, 36]. It is important to note that none of the studies included in the cited meta‐analyses have reported the incidence or severity of preoperative PF cartilage lesions, so their influence on the subsequent risk of PF‐OA is not known [35, 36, 48]. Histologic analysis of human cartilage after TP revealed an overall unimpaired chondrocyte viability [52]. Dejour et al. recently reported on a case series of 44 thick‐flap TPs and found good clinical results after an average of 14.8 years, but only minimal signs of radiographic degenerative changes in 62% of all knees [17]. Nevertheless, concerns remain that the procedure of TP may be associated with progressive cartilage deterioration [33, 34, 52]. They were supported by a recent finite element (FE) analysis that proposed an increase in retropatellar contact pressures and a decrease in the PF contact area after TP [33]. In contrast, results of a cadaveric biomechanical study suggest that high‐grade TP itself is responsible for increased contact pressures, while decreasing both stability and contact area in the PFJ [25].

One explanation for the divergence may be the consideration of patellar geometry in order to achieve PF congruence [3, 38]. Controversely, neglecting the patellar shape may result in failure to achieve these desired effects [33]. In a case series of 18 TPs, Banke et al. found significant improvements in both PF positional parameters and functional outcome but no signs of early PF‐OA [7]. In this study, the trochlear shape and resection depth were planned on preoperative MR images. At the same time, several studies have identified TD as a major RF (odds ratio 15.4) for PF cartilage deterioration and early PF‐OA [28, 31, 49]. Accordingly, we found a very high incidence of advanced cartilage lesions in the analysed cohort, which consisted exclusively of high‐grade TP. We also found a negative correlation between patient age at the time of surgery and the AMADEUS score. These observations support the findings of other studies that have observed an association between TD and PF cartilage deterioration over time [28, 31]. At the final follow‐up, however, there was no evidence of progressive cartilage deterioration, but an unexpected increase in the AMADEUS score. This may be explained by an unloading effect of the affected chondral lesions, allowing the formation of cartilage repair tissue, thus improving their radiologic appearance in terms of the AMADEUS scoring tool (Figure 7). Ultimately, these results should not suggest chondral restoration but are consistent with constant PF cartilage integrity rather than progressive deterioration. Certainly, it is important to note that these are short‐term observations that cannot provide certainty about the long‐term risk of PF‐OA. Given the high incidence of primary cartilage lesions, it can be assumed that TP may prolong but not halt the development of PF‐OA, while there is no evidence of an accelerated onset. Of course, other (technical) considerations could potentially affect the PF cartilage after TP. According to a meta‐analysis, Dejour's V‐shaped technique bears a higher risk of PF‐OA, when compared to Bereiter's thin‐flap technique or Goutallier's recession technique [36]. Furthermore, using a high‐speed burr may cause more thermal damage to the thin chondral flap compared to the use of osteotomes.

Despite the large number of cases included, we had to accept a relatively high number of patients loss to follow‐up, mainly due to unknown changes in contact data or incomplete radiologic follow‐up. A high loss to follow‐up rate poses the risk of an inaccurate outcome analysis with an increased risk of missing poor outcomes. One possible explanation for this could be the young average age of patients who are likely to move as part of their training or studies. Another reason could be the large catchment area. Patients travelling from other parts of the country for surgical care only did not participate in regular radiologic follow‐up. However, this is offset by complex clinical and radiological data collection, which in our view compensates for this limitation. At the same time, similar lost‐to‐follow‐up rates of around 22%–58% are reported in the literature [39, 44, 47].

Of course, the results of our study must be interpreted in the context of a highly preselected patient cohort, which differs from the distributions commonly reported in the literature. Specifically, while previous studies suggest that up to 80% of patients with patellar instability present with at least one or two anatomical RFs, our cohort demonstrated a different pattern [2]. On the one hand, this may result from the strict inclusion and exclusion criteria of our study, which led to the exclusion of a large proportion of patients with relevant extraarticular RFs (Figure 3). Another possible explanation for this discrepancy may be the high specialisation and experience of the senior surgeon, which likely influenced patient selection. Yet, the identification and quantification of anatomical RFs depend strongly on the specific definitions and thresholds applied. In this regard, the literature demonstrates significant heterogeneity in these definitions and cutoffs, making direct comparisons across studies challenging [1, 2, 4, 5, 26, 50]. This variability may also partly account for the observed differences between our findings and previously reported data.

### Limitations

There are some limitations to this study. First, this study reports on a short‐term follow‐up that allowed analysis of early cartilage deterioration, but not the development of PF‐OA. Second, even though the included patients are very likely a representative collective, the high loss to follow‐up rate remains a limitation of this study. Another limitation was the lack of pre‐ and postoperative radiologic measurements of patellotrochlear congruency. Furthermore, no control group analysis was performed, especially regarding the influences of TD and TP on PF cartilage. Finally, there was a certain degree of pre‐selection of the study collective, due to a high level of expertise of the main surgeon.

## CONCLUSION

Sulcus‐deepening TP with MPFL‐R and LRL yields good to excellent short‐term results without progressive chondral deterioration, enabling patients to return to their prior or higher activity levels despite advanced preoperative chondral lesions. TP can be considered a safe, joint‐preserving technique for patellofemoral stabilisation.

## AUTHOR CONTRIBUTIONS

Jannik Frings and Arno Schmeling conceptualised and designed the study. Jannik Frings and Eva Janßen performed the data acquisition. Jannik Frings and Eik Vettorazzi performed the statistical analysis. Eik Vettorazzi provided statistical consultation. Jannik Frings wrote the manuscript. Jannik Frings and Arno Schmeling created the graphs and figures. Matthias Krause, Karl‐Heinz Frosch and Andreas Weiler revised the manuscript. All authors reviewed, refined and approved the final manuscript.

## CONFLICT OF INTEREST STATEMENT

Karl‐Heinz Frosch receives royalties from Arthrex (Naples, FL, USA). Karl‐Heinz Frosch and Matthias Krause receive honoraria for lectures from Arthrex (Naples, FL, USA). Andreas Weiler receives royalties from Medacta International (Castel San Pietro, Switzerland) and honoraria for lectures from Enovis (Freiburg, Germany). Arno Schmeling receives payments from a consulting contract with Arthrex (Naples, FL, USA) and honoraria for presentations from Conmed (Utica, NY, USA). He is head of the Patellofemoral Committee of the German Society for Knee Surgery. Jannik Frings and Eva Janssen declare no conflicts of interest.

## ETHICS STATEMENT

The study was approved by the local ethics committee (PV5191).

## Data Availability

All data generated or analysed during this study are included in this published article.
